# Laparoscopic orchidopexy for the treatment of cryptorchidism in adults: a description of the technique and outcomes

**DOI:** 10.1186/s12894-023-01386-4

**Published:** 2024-01-02

**Authors:** Hu Han, Jiaxing Li, Hong-en Lei, Hang Yin, Long Tian

**Affiliations:** 1 Department of Urology, Beijing Chao-Yang Hospital, Capital Medical University, 8 Gong Ti Nan Road, Beijing, 100020 China; 2 Institute of Urology, Beijing Chao-Yang Hospital, Capital Medical University, Beijing, China

**Keywords:** Cryptorchidism in adults, Laparoscopic orchidopexy, Reproductive hormones, Tension-free hernia repair

## Abstract

**Background:**

There are few studies on cryptorchidism in adults, and its treatment is still controversial.

**Methods:**

To summarize the surgical strategy and clinical efficacy of laparoscopic orchidopexy for the treatment of cryptorchidism in adults, 37 adult cryptorchidism patients were retrospectively analyzed between September 2017 and February 2022. All 37 patients underwent laparoscopic orchidopexy, of whom 33 underwent inguinal hernia repair without tension. The intraoperative procedures and surgical techniques were recorded in detail. Preoperative examination and regular postoperative review of color Doppler ultrasound, and reproductive hormone, alpha-fetoprotein, human chorionic gonadotropin, and lactate dehydrogenase levels were performed.

**Results:**

All testes descended successfully into the scrotum, including 25 through the inguinal route and 12 through Hesselbach’s triangle route. No intraoperative or postoperative complications were observed. The follow-up time was 38.6 (± 19.4) months, and no evidence of testicular malignancy was found during the follow-up period. After analyzing the reproductive hormone levels at 1 year postoperatively in 28 patients with more than 1 year of follow-up, it was found that the patients had a significant increase in testosterone levels and a decrease in follicle-stimulating hormone levels after surgery. None of the patients showed any significant improvement in semen quality after surgery.

**Conclusion:**

Our study suggests that laparoscopic orchidopexy is a safe and feasible surgical procedure for the treatment of cryptorchidism in adults, especially high cryptorchidism, which is difficult to treat. After comprehensive consideration, preserving the testis should be preferred for treating cryptorchidism in adults to maximize the protection of the patient’s reproductive hormone secretion function.

## Introduction

Cryptorchidism or undescended testis (UDT) is the most common congenital genitourinary tract anomaly, with a prevalence of approximately 3.4–5.8% in term infants and approximately 30% in preterm infants [1]. Cryptorchidism of one or both testes can occur. It can be classified as retractable testis, high intra-abdominal cryptorchidism, inguinal canal cryptorchidism, and high scrotal cryptorchidism depending on the location of the testis. The causes of cryptorchidism are multifaceted and include genetic, hormonal, environmental, lifestyle, and maternal factors [2]. Cryptorchidism is usually diagnosed and treated in childhood, and adult patients with cryptorchidism are relatively rare. Current literature reports on treating cryptorchidism in adults are scarce, and most are case reports [3–6]. In this study, we investigated the safety and efficacy of laparoscopic orchidopexy for the treatment of cryptorchidism in adults to highlight the technique and outcomes.

## Patients and methods

This retrospective study was approved by the Ethics Committee of Beijing Chaoyang Hospital, Capital Medical University. The inclusion criteria for this study were as follows: [1] adult males, [2] patients with unilateral/bilateral cryptorchidism, and [3] patients with a strong desire to procreate or a strong desire for testicular preservation. The exclusion criteria in this study were as follows: [1] significant testicular atrophy revealed through laparoscopic exploration; [2] undescended testes with evidence of deterioration; [3] comorbid severe cardiovascular disease, diabetes mellitus, liver dysfunction, renal disease, and severe hematological disorders; and [4] contraindications to general anesthesia. We identified 37 adult cryptorchidism patients aged 30.92 ± 9.1 years between November 2017 and February 2022 (Table 1). Reasons for consultation included undiagnosed cryptorchidism (n = 14), oligoasthenospermia (n = 3), and azoospermia (n = 10). One patient had obstructive azoospermia; the remaining 9 patients had nonobstructive azoospermia, including one with combined Kalman’s syndrome and one patient with a chromosomal examination result of 46xy,21pstk+). Failure of testis descent under open surgery (n = 5), inguinal hernia (n = 3), sudden inguinal region pain (n = 1), and contralateral testicular tumor (n = 1) were observed. Of the 37 patients, 29 had unilateral cryptorchidism, 8 had bilateral cryptorchidism, and 25 were diagnosed with a combined inguinal hernia by preoperative imaging. Thirty-two of the 37 patients had inguinal cryptorchidism (the undescended testis was not palpable in 20 patients and was palpable in 12 patients on specialist examination), and 5 patients had high cryptorchidism. The specific surgical steps and surgical techniques were recorded in detail, the intraoperative testicular descent pathway was recorded, intraoperative and postoperative surgical complications were recorded, and the patients were followed up for recurrence and other uncomfortable symptoms, emphasizing dynamic observation for malignant changes. Each surgery was performed by the same team of surgeons. SPSS 22.0 statistical software was applied for the statistical analysis, a paired t test was used to compare each test index before and after surgery, and *P* < 0.05 was considered to indicate a significant difference.

**Table 1:** Patients’ Clinical Character

Demographics (N = 37 patients)	
Age/ Course (years)	30.9 ± 9.1
BMI ( kg/m^2^)	25.2 ± 3.6
Hospital stay(d)	7.4 ± 1.8
Follow-up time(month)	38.6 ± 19.4
Unilateral / Bilateral cryptorchidism, n (%)	
Unilateral	29(78%)
Bilateral	8(22%)
Location of Cryptorchidism	
high cryptorchidism	5(14%)
inguinal cryptorchidism	32(86%)
Testicular descent pathway, n (%)	
Original channel Hesselbach’s triangle	25(68%) 12(32%)
Operative time(min)	
Unilateral LO and LTIHR	162.2 ± 41.6
Bilateral LO and LTIHR	235.8 ± 70.2

BMI = body mass index, LO laparoscopic orchidopexy, LTIHR = laparoscopic tension-free hernioplasty. Categorical variables are presented as number (%). Continuous variables are presented as mean ± standard deviation

### Preoperative preparation

All patients were examined preoperatively using color Doppler ultrasonography (DUS) or abdominal magnetic resonance imaging (MRI) to detect the volume and location of the testes. Preoperative laboratory tests included reproductive hormone, alpha-fetoprotein (AFP), human chorionic gonadotropin (hCG), and lactate dehydrogenase (LDH) level tests, in addition to tests regarding biochemistry, liver function, coagulation function, and infectious diseases. Semen analyses were performed on patients with fertility intentions.

### Surgical technique

#### Establishment of the surgical cavity

After successful anesthesia, the patient was placed in a head-low, foot-high position. An artificial pneumoperitoneum was established with a pneumoperitoneal pressure of approximately 12 mmHg. A 10-mm Trocar was placed at the navel, and 10- and 5-mm Trocars were placed at the external edges of the right and left rectus abdominis muscles, respectively.

#### Intraperitoneal exploration

The inguinal canal was explored laparoscopically. If the preoperative examination suggested that the testis was in the inguinal canal, the peritoneum was incised above the deep inguinal ring with an extended curved incision, and the testis was searched for in the inguinal canal. If the preoperative examination suggested high cryptorchidism (a testis located above the deep inguinal ring), then the testis was searched for above the inner inguinal orifice. After finding the testis, the size of was observed, and any apparent abnormalities in morphology were noted. The vas deferens was searched for in the medial pelvic vas deferens area. In our experience, very few adult patients with cryptorchidism have comorbid vas deferens dysplasia. If it was difficult to find obvious vas deferens-like structures intraoperatively, careful exploration was performed to determine whether the dysplastic vas deferens was fibrous-stripe-like and without a lumen.

#### Orchidopexy

**Fig. 1:**
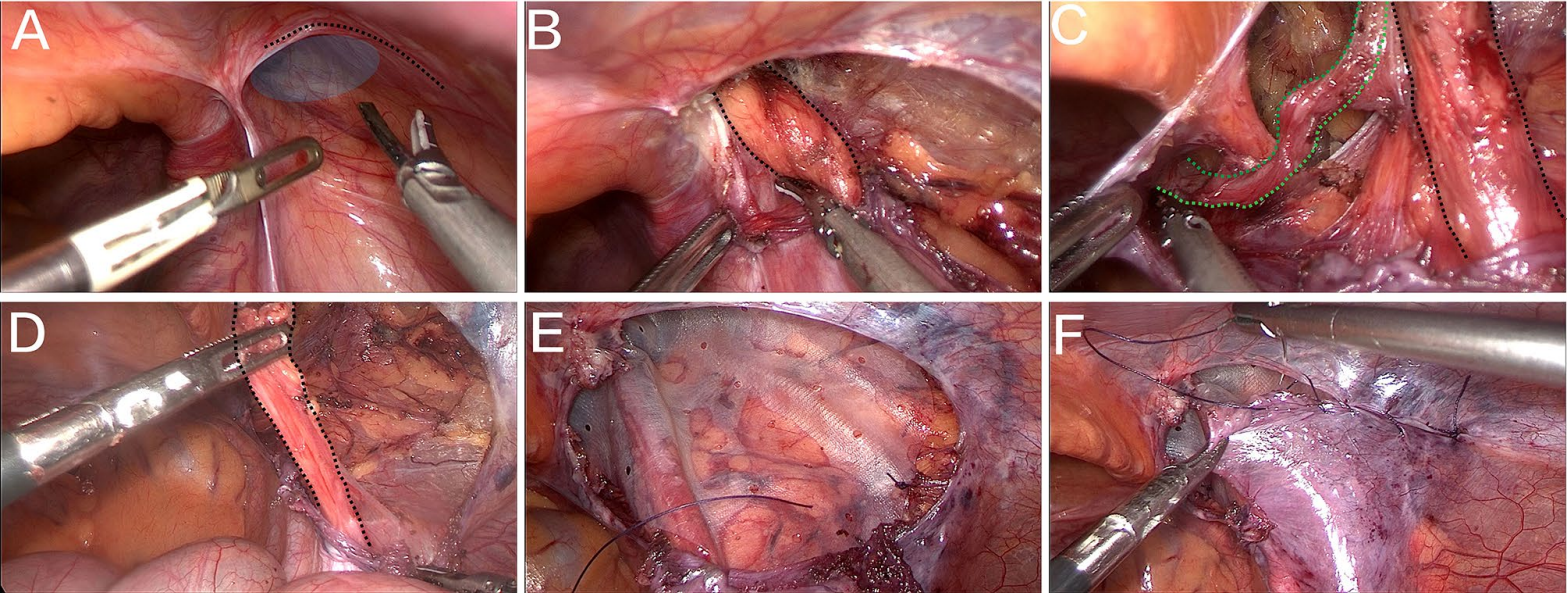
**A**: Opening the peritoneum with an arched incision above the deep inguinal ring (the black dashed line shows the incisional alignment, and the blue shaded area shows the unclosed internal inguinal canal ring). **B**: Separating the spermatic cord (black dashed line shows the spermatic cord). **C**: Fully freeing the medial vas deferens (the green dashed line shows the vas deferens, black dashed line shows the spermatic cord). **D**: Fully freeing the retroperitoneal spermatic cord (black dashed line shows the spermatic cord). **E**: Suturing to fix the patch **F**: Absorbable continuous sutures to close the peritoneum

The peritoneum was opened in an arc above the deep inguinal ring to expose the preperitoneal space (Fig. 1A). The fibrous connective tissue on the spermatic cord was carefully separated by combining blunt and sharp separation to loosen the spermatic cord (Fig. 1B). The vas deferens was searched for in the medial pelvic section of the deep inguinal ring, and the vas deferens was entirely freed (Fig. 1C). Care was taken to protect the vas deferens and the inferior epigastric vessels during separation. After the spermatic cord and the vas deferens were entirely released, the testis could be placed into the scrotum through the original channel without tension (Fig. 1D). If the patient was had high cryptorchidism or had high tension during descent, the channel was re-established at Hesselbach’s triangle of the inferior epigastric vessels, and the length of the descending channel was shortened so that the testis could be placed into the scrotum without tension.

The scrotal skin and scrotal dartos were incised in the middle of the scrotum. Vascular forceps were utilized to create a gap between the scrotum and sarcoid with sufficient dimensions to accommodate the testicle. Through this established gap, an atraumatic oval forceps was introduced, guided by laparoscopic observation and entering Hesselbach’s triangle or the inguinal canal via the external inguinal ring. Subsequently, atraumatic oval forceps were employed to secure the chorda gubernaculum, facilitating the descent of the undescended testis from the abdominal cavity to the scrotum. Wet gauze was applied around the spermatic cord to ensure hermetic sealing of the pneumoperitoneum. If the testis was small, a small amount of tissue was biopsied after descent into the scrotum by incising the tunica albuginea. After ensuring that the spermatic cord was torsion-free, the tunica vaginalis was secured to the scrotal dartos and fixed using interrupted sutures with silk thread to prevent retraction of the testis. After placing a rubber drainage sheet at the lowest scrotal incision, the scrotal skin incision was closed layer by layer.

#### Tension-free hernioplasty

Tension-free hernia repair was performed simultaneously if the patient had a combined inguinal hernia. The hernia sac was separated, and the Biodesign Inguinal Hernia Graft (Cook Medical, USA) was inserted and spread sufficiently in the anterior peritoneal space to cover the area of the internal ring opening and Hesselbach’s triangle area (Fig. 1E). The mesh was secured with sutures to prevent displacement. The peritoneum was closed with continuous sutures using absorbable sutures (Fig. 1F).

## Results

Thirty-seven patients underwent successful laparoscopic orchidopexy, and 32 underwent simultaneous laparoscopic tension-free hernia repair (7 underwent prophylactic tension-free hernioplasty). The operative time for unilateral testis descent fixation was 162.0 (± 41.6) min, and that for bilateral testis descent fixation was 235.8 (± 70.2) min. The hospitalization time was 7.4 (± 1.8) d (Table 1). No intraoperative vascular injury or bleeding occurred during the operation, and no patients were converted to open surgery. Postoperative complications were scored using the Clavien‒Dindo score [7]. Grade I complications were observed in 8 patients, mainly postoperative pain, which generally resolved significantly 2–3 days after surgery. No grade II, III, or IV complications were observed, and no complications, such as wound infection or hydrocele, occurred. Follow-up included male reproductive system ultrasound and reproductive hormone, AFP, hCG, and LDH level tests. The follow-up time was 38.6 (± 19.4) months for a maximum of 65 months. No evidence of testicular malignancy was found during the follow-up period, and no abnormalities were observed on tumor marker examination or ultrasonography. One patient had postoperative testicular atrophy, and no significant testicular atrophy was found in the rest of the patients after surgery.

In analyzing the data of reproductive hormone levels at 1 year postoperatively in 28 patients with more than 1 year of follow-up (5 patients had the relevant tests completed at the local hospital, and we were unable to obtain complete follow-up data during the follow-up process), we observed a significant increase in testosterone levels and a decrease in FSH levels after surgery (Table 2). Among the 13 patients with cryptorchidism who visited the clinic for oligoasthenospermia or azoospermia, one patient’s spouse conceived spontaneously, and three patients’ spouses gave birth after assisted reproductive technology treatment. None of the patients showed significant improvement in semen quality after surgery.

**Table 2:** Comparison of preoperative and postoperative (N = 28)

Index	Pre-operation	Post-operation	P value
TT(ng/mL)	3.21 ± 2.17	4.42 ± 2.64	0.0133
FSH(IU/L)	8.71 ± 6.89	7.87 ± 4.80	0.1374
LH(IU/L)	4.86 ± 3.75	4.82 ± 2.47	0.9353
E_2_(pg/ml)	35.93 ± 12.41	45.25 ± 27.22	0.0527
PRL(ng/ml)	11.32 ± 6.98	9.65 ± 3.82	0.1912
Testicular volume(cm^3^)	4.39 ± 2.54	4.69 ± 2.70	0.5889

TT = testosterone, FSH = follicle stimulating hormone, LH = Luteinizing hormone, E2 = estradiol, PRL = prolactin. TT < 0.2 ng/mL is calculated as 0. Continuous variables are presented as mean ± standard deviation

## Discussion

The current mainstream treatment modalities for cryptorchidism include laparoscopic surgery and open surgery. Although the operation time of laparoscopic surgery is longer than that of open surgery, there is not sufficient evidence to confirm that laparoscopic surgery is superior to open surgery in terms of intraoperative bleeding, complications, and long-term prognosis [8]. However, according to our treatment experience, laparoscopic surgery for cryptorchidism in adults has the following advantages: (1) Laparoscopic orchidopexy is feasible with laparoscopic exploration, which enables fine visualization of anatomical structures. (2) Laparoscopic orchidopexy substantially reduces surgical trauma. (3) Laparoscopic surgery for cryptorchidism in adults can simultaneously complete the descent of the testis and hernia repair in one stage without additional incisions. (4) Laparoscopic surgery for cryptorchidism in adults can sufficiently free the vas deferens and spermatic cord blood vessels. The vas deferens can be released upward along the spermatic cord to reduce the tension during descent.

For patients with palpable cryptorchidism in the inguinal region, laparoscopic orchidopexy can be considered a viable surgical option. Among the 12 patients with palpable inguinal cryptorchidism, 5 sought treatment in adulthood due to unsuccessful descent of the undescended testis during childhood. In these patients, the testis was high, the adhesions were severe, the anatomical level was unclear, and open surgery would be extremely difficult. However, the spermatic cord could be entirely freed under laparoscopy, up to the level of the lower pole of the kidney, so that the high testis could descend through laparoscopic surgery, avoiding forced cryptorchidectomy because the length of the spermatic cord was not long enough to reach the testis. The remaining 7 patients, all young males, expressed a strong preference for minimally invasive surgery to mitigate the aesthetic impact of a large incision or a fervent desire for testicular preservation. After thorough consideration of the advantages and disadvantages, laparoscopic orchidopexy was conducted for these 7 patients.

**Fig. 2:**
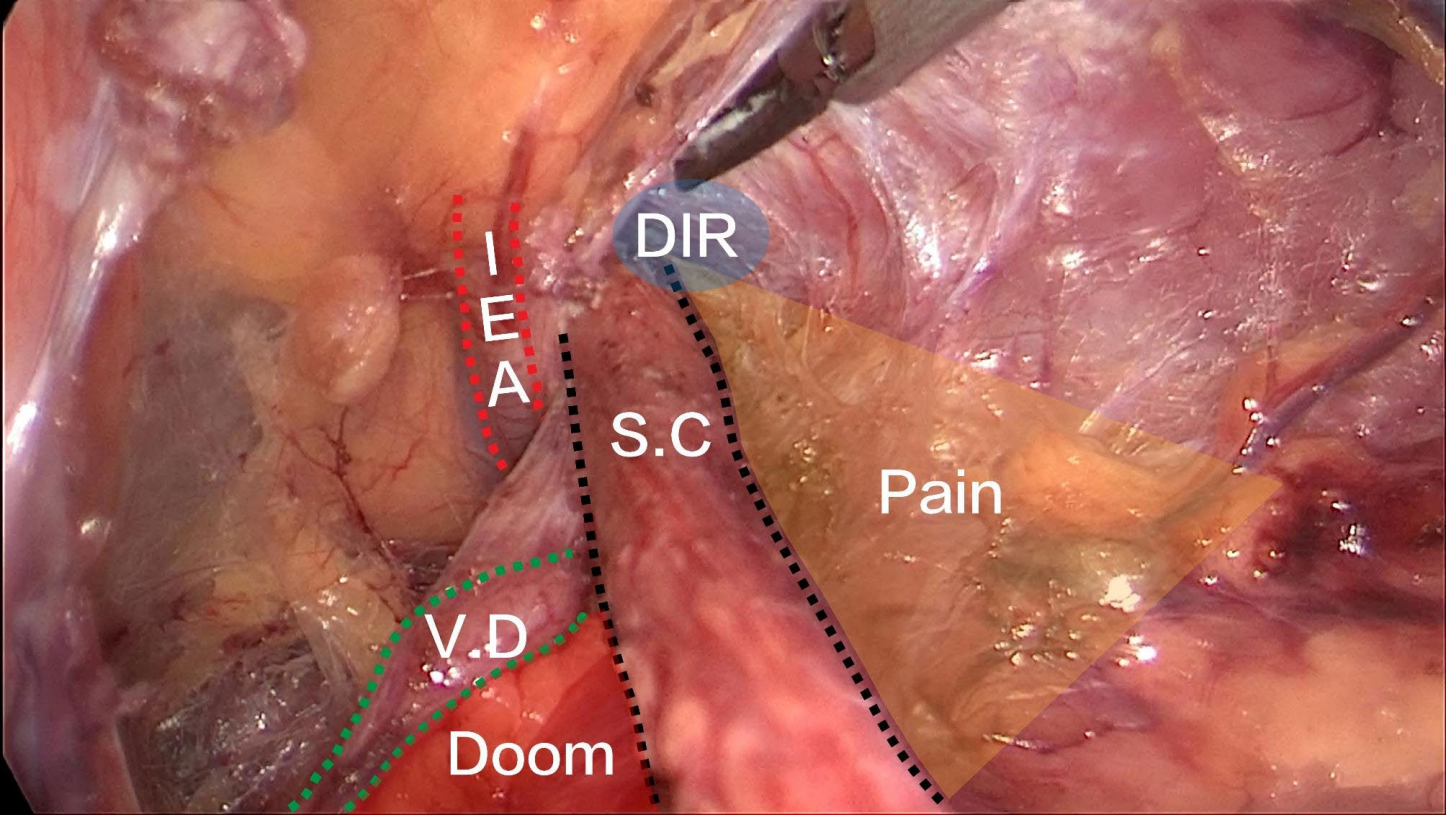
Anatomical landmarks in laparoscopic orchidopexy: The yellow shaded area is the “pain triangle”. The red-shaded area is the “danger triangle”, which contains the external iliac artery and the femoral nerve. The blue-shaded area is the deep inguinal ring (DIR). The red dashed line is the inferior epigastric artery (IEA), the green dashed line is the vas deferens (VD), and the black dashed line is the spermatic cord (SC)

To ensure the results of this procedure, the following points should be noted intraoperatively: (1) The triangle formed between the spermatic cord and the vas deferens, known as the “danger triangle” (Fig. 2), should be identified [9]. When separating the spermatic cord in this area, care should be taken to avoid injury to the external iliac vein and external iliac artery. (2) The “corona mortis” vessel should be located on the medial side of the vas deferens, which mainly travels on the surface of the rami superior ossis pubis and enters the obturator inwardly or anastomoses with the obturator vessel. Its injury should be avoided during surgery. (3) Attention should be given to the “pain triangle” during the lateral freeing of the spermatic cord to avoid injury to the femoral branch of the genitofemoral nerve and femoral nerve [9]. In this study, none of the patients experienced scrotal pain at follow-up. (4) The patch should be fully spread in the anterior peritoneal space. (5) Care should be taken to restore the original structure of the peritoneum during suturing to avoid the formation of adhesions due to contact between the intestinal canal and the patch.

Cryptorchidism is a recognized risk factor for testicular germ cell tumors (TGCTs) [10]. The potential risk of testicular malignancy in patients with cryptorchidism after testicular fixation is 2.9 times the baseline prevalence in adult men [11, 12]. The incidence of testicular tumors in patients with cryptorchidism is 5 to 10 times higher than that in the population without cryptorchidism [13, 14]. Reports on the potential risk of testicular cancer in cryptorchidism patients have focused on the pediatric urology field. In contrast, few studies have reported testicular malignancy after testicular descent and fixation in adults. The previous concept is that cryptorchidism in adults should be treated by resection as much as possible, but according to our treatment experience, we have developed some new opinions. The preserved testis can continue to produce sex hormones and maintain the level of sex hormones in the patient’s body, which greatly ensures the patient’s quality of life after surgery [15]. In our study, we found that this procedure significantly increased blood testosterone levels in patients after surgery, and there was a trend toward lower postoperative serum FSH levels compared with preoperative levels (Table 2). No evidence of malignancy was found in the 37 adult patients with cryptorchidism who underwent testicular descent and fixation at a maximum follow-up time of 63 months, no abnormalities were found in tumor marker tests or on ultrasonography, and only one patient showed significant testicular atrophy. Therefore, we believe that various considerations are needed in determining whether this procedure should be performed, and we prefer to preserve the testis to protect the reproductive hormone secretion function of patients to the greatest extent.

At the same time, the following points should be noted to minimize adverse prognosis in patients due to postoperative testicular malignancy: (1) Intraoperatively, an incisional biopsy should be performed after the descent of a small testis. (2) For the testes with obvious atrophy and evidence of deterioration, the testis should always be removed. Among the 43 adult patients with cryptorchidism seen at our center from 2017 to 2023, 6 patients did not have indications for preserving the testis. Therefore, we treated these six patients with laparoscopic orchidopexy. (3) Attention should be given to full preoperative communication with patients and families about the risk of postoperative testicular atrophy and malignant transformation. The importance of regular postoperative review should be emphasized. Patients should be educated to regularly perform self-examinations after surgery, including whether atrophy, retraction, hardening, and sudden enlargement of the testis occurs. (4) Patients should be checked every 3–6 months in the first year and annually from the second postoperative year. The check should include color Doppler ultrasound of the male reproductive system and reproductive hormone, AFP, hCG, and LDH level tests to detect testicular malignancy early and treat it in time.

In adult patients with cryptorchidism who strongly desire to have children, the recovery of spermatogenic function after descent and fixation is limited according to our treatment experience and previous related studies [15]. Elevated intraperitoneal temperature inhibits spermatogonial differentiation [16, 17]. A 5% increased risk of needing future assisted reproductive treatment was observed for every 6-month delay in performing the procedure after 18 months of life [18, 19]. In our study, we found that among 13 patients with cryptorchidism who were seen for oligozoospermia or azoospermia, one patient’s spouse conceived spontaneously, and three patients’ spouses gave birth after assisted reproductive technology treatment. In contrast, the semen quality of the remaining patients did not show significant improvement after surgery. We recommend microTESE (microsurgical testicular sperm extraction) if no significant improvement is seen in the semen routine examination 6 months after surgery.

Cryptorchidism combined with inguinal hernia accounts for 25.6% of all cryptorchidism cases [20]. Of the 37 patients, 32 underwent a concomitant hernia repair. Hernia prophylaxis is essential to ensuring postoperative outcomes, and 7 patients underwent prophylactic tension-free hernioplasty. Pediatric urologists believe that intraoperative nonclosure of the internal ring opening does not cause postoperative inguinal hernia [21]. However, this concept may not be applicable in the treatment of cryptorchidism in adults, and we recommend prophylactic tension inguinal hernia repair in the following two cases. First, when descending the testis through the Hesselbach triangle, the original channel forms a cleft. Second, when descending the testis through the original inguinal channel, the inguinal ring is observed to be too spacious intraoperatively. Regarding the choice of patch material, we recommend using biologic mesh for young people with fertility intentions to reduce the stretching and irritation of the spermatic cord.

The shortcomings of this study are as follows: (1) This was a retrospective study because the prevalence of cryptorchidism in adults is low, and it is challenging to conduct randomized controlled trials. (2) The number of patients in this study was small, and studies with larger sample sizes are needed to confirm this surgery’s feasibility and long-term clinical efficacy. (3) No evidence of malignancy was found in preoperative imaging examinations, and whether cryptorchidism requires routine pathological examinations should be further explored after increasing the sample size.

## Conclusion

Our study suggests that laparoscopic treatment of adult cryptorchidism is safe and effective and has a good prognosis. We recommend that the treatment of adult cryptorchidism should be based on the patient’s age, cryptorchidism classification, cryptorchidism developmental status, and reproductive history and that preserving cryptorchidism should be preferred to maximize the protection of reproductive hormones. Tension-free hernioplasty is used for patients with a combined inguinal hernia, and patients without a combined inguinal hernia are considered for prophylaxis with a mesh depending on the situation.

## Data Availability

The data that support the findings of this study are available from the corresponding author upon reasonable request.
